# The Toronto declaration on bridging knowledge, policy and practice in aging and disability: Toronto, Canada, March 30, 2012

**DOI:** 10.5334/ijic.1086

**Published:** 2012-11-16

**Authors:** Jerome Bickenbach, Christine Bigby, Luis Salvador-Carulla, Tamar Heller, Matilde Leonardi, Barbara LeRoy, Jennifer Mendez, Michelle Putnam, Andria Spindel

We, as organizers and participants of the 2011 Growing Older with a Disability (GOWD) Conference, a part of the Festival of International Conferences on Caregiving, Disability, Aging and Technology (FICCDAT), held in Toronto, Canada June 5–8, 2011 forward this declaration and invite governmental, non-governmental, professional, and consumer stakeholders to join us in supporting and implementing this plan of action.

The 2011 World Report on disability, produced jointly by the World Health Organization (WHO) and the World Bank, estimates that there are over one billion people with disabilities in the world today, of whom nearly 200 million experience significant difficulties. At the same time, in almost every country, the proportion of people aged over 60 years is growing faster than any other age group, forecast to reach 1.5 billion by 2050, according to the Global Health and Aging Report, also released in 2011 by WHO in partnership with US National Institute on Aging. This means that in the years ahead disability will be an even greater concern to developed and developing nations due to aging populations, higher risk of disability in older people, as well as the global increase in chronic health conditions, such as diabetes, cardiovascular disease, cancer and mental health disorders. Taken together, the dual phenomena of global aging and increased longevity for individuals with disabilities represent significant advances in public health and education.

However, along with these positive trends come new challenges for the 21st century. These include: strains on pension and social security systems; preparing health providers and societies to meet the needs of populations aging with and aging into disability; preventing and managing age and disability associated secondary conditions and chronic diseases; designing sustainable policies to support healthy aging and community-living as well as long-term and palliative care; and developing disability and age-friendly services and settings.

Bridging the fields of aging and disability research, policy, and practice is critical for meeting these challenges. All of us aspire to healthy aging, regardless of the presence of age-related impairments or disabling conditions. The experience of growing older with a disability and growing older into a disability may differ—in part because of the different dynamics of ageism and ableism and the differences in economic and social conditions that result—but these life course trajectories present similar challenges and opportunities. In this document we seek common ground, in terms of the modern conception of active aging and of disability, defined as difficulty in functioning at the body, person or societal levels experienced by an individual with a health condition in interaction with the person’s physical, social and attitudinal environment. Moreover, we firmly believe that, despite the distinctions between aging and disability created by professionals, academics, advocacy NGOs, public policies and government agencies, the time has come to emphasize similarities in experiences and needed supports, services and policies rather than focusing on differences. Distinctions between early and late onset of disability are to a large extent a reflection of policy issues—with various utilities across nations—but they do provide a picture of the parameters of practice and research that can inform bridging and consequences of this distinction.

This declaration builds upon the Barcelona Declaration on Bridging Knowledge in Long-Term Care and Support, March 5–7, 2009, the Graz Declaration on Disability and Ageing, 9th June, 2006, the Linz Declaration as well as United Nation’s Conventions (in particular the United Nations Convention on the Rights of Persons with Disabilities and the United Nations 2002 Political Declaration from the Madrid World Assembly on Aging II) and international directives that recognize the human rights and the biopsychosocial approach to disability. Bridging encompasses a range of concepts, tasks, technologies and practices aimed at improving knowledge sharing and collaboration across stakeholders, organizations and fields in care and support for persons with disabilities, their families, and the aging population. Bridging tasks include activities of dissemination, coordination, assessment, empowerment, service delivery, management, financing and policy. The overall purpose of bridging is to improve efficiency, equity of care, inclusion and support at all levels, from the person to the society. It is also an issue of recognition of the complexity of the human condition from birth to death, the capabilities of all people, and the need for a conceptual vision that takes into consideration planning for a society where participation of all citizens is the ultimate goal.

## Based on the findings of the GOWD and larger FICCDAT meetings, we assert that:

- **National and international bridging of aging and disability knowledge, policy and practice must be actively promoted.** Aging with and aging into disability are global population trends. Cross-national and international collaborations can support effective and efficient knowledge development and transfer, implementation of best practices, and facilitate information exchange among and empowerment of persons with disabilities and their families.
- **Bridging is composed of several activities which must occur simultaneously, at multiple levels of knowledge development, policy and practice, and include disability and aging stakeholder groups.** The scope of required bridging activities is broad, including the analysis of public policies, interdisciplinary research, the development of professional best practices, and coalition building across advocacy groups and among individual stakeholders. Older adults and people with disabilities and their families must be meaningfully included in bridging activities in recognition of their rights to self-determination and social inclusion.
- **Building effective bridges across aging and disability knowledge, policy and practice requires interdisciplinary collaboration and engagement with national and international decision-makers.** Development of effective models of bridging and successful bridging practices requires engagement of professional and citizen stakeholders bringing together relevant knowledge and experience. Decision leaders must engage knowledge brokers to pursue program and policy changes that support bridging activities.
- **Connecting the field of aging and disability will require development of a clear model of bridging.** Research at all levels will support the science of bridging as it develops. However, research must give immediate and persistent attention to the pace of bridging to assure that it aligns with the needs of the person aging with disability in order for them to negotiate and make life choices, navigate support and service systems, and engage in opportunities for full inclusion and participation in society.
- **Bridging requires developing a common terminology and knowledge base.** Tasks include activities of dissemination, coordination, assessment, empowerment, service delivery, management, financing and policy. Technologies include various Information Technologies, assessment instruments and guidelines. Bridging practices should be catalogued and incorporated to open-access repositories for use by aging and disability networks.

## Therefore we identify the following priority areas for bridging aging and disability knowledge, policies, and practice:

### Health and well-being:

Improved access to healthcare services; improved diagnosis and treatment of secondary conditions and diseases; care coordination; health literacy; health promotion and wellness; prevention of age-related chronic conditions; prevention of abuse and neglect; reduction in pre-mature mortality and training of health professionals in aging and disability.

### Inclusion, participation and community:

Accessible societies, including age and disability friendly communities, removal of barriers of any kind: architectural, cultural, legislative. Impact and implications of aging and disability on civic and community engagement, and the role of technology and universal design in fostering inclusion, participation and knowledge management.

### Long-term supports and services:

Support for families and caregivers, training and education of direct support professionals; self-determination, access, availability, and affordability of supports and services; ethical issue related to non-discrimination, such as in palliative care, end of life issues.

### Income security:

Employment, retirement security, asset development; accommodation and accessibility in the work setting; value of non-paid social and community contributions.

### Science of bridging:

Research on bridging aging and disability and on ways to transfer this knowledge locally, nationally, and internationally to policy development.

## We therefore recommend that:

An international agenda for bridging aging and disability be formally developed through the involvement of researchers, practice professionals, policy-makers, older adults, persons with disabilities and their families.

Public and private funders provide financial support for research and scholarship that advances the science of bridging aging and disability knowledge, practice and policies.

Health and social policy-makers incorporate bridging and knowledge transfer as key strategies in policy planning for building a society where all citizens can fully participate including persons with disabilities of all ages.

## We invite endorsement and implementation of this declaration

The authors, all of whom were participants at the Growing Older with a Disability conference at FICCDAT 2011, endorse this Declaration and invite feedback.

Response can be sent to the attention of Toronto Declaration@marchofdimes.ca

Individuals and organizations which have endorsed this declaration are listed below. Others are invited to add their endorsement by sending an email with your full contact information to TorontoDeclaration@marchofdimes.ca, adding *‘TD Endorsement’* in the subject line.

Most importantly, we call upon governments, practitioners, policy-makers and academics to work together with consumers and their families to ensure attention and implementation of the preceding recommendations.

Co-Chairs of Growing Older with a Disability (GOWD) conference, Festival of International Conferences on Caregiving, Disability, Aging and Technology (FICCDAT), 2011

Margaret Campbell, PhD, Jennifer Mendez, PhD, Sandy Keshen

## Endorsement by the Authors

**Jerome E. Bickenbach, PhD**

Department of Health Sciences and Health Policy, University of Lucerne and Schweizer Paraplegiker-Forschung, Nottwil, Switzerland

**Christine Bigby, PhD**

Department of Social Work and Social Policy, La Trobe University, Bundoora, Victoria, Australia

**Luis Salvador-Carulla, MD PhD**

Faculty of Health Sciences, University of Sydney, Sydney, Australia

**Tamar Heller, PhD**

Rehabilitation Research and Training Center on Aging with Developmental Disabilities, University of Illinois at Chicago, Chicago, IL, USA

**Matilde Leonardi, PhD**

Head Neurology, Public Health, Disability Unit Foundation IRCCS Istituto Neurologico Carlo Besta, Milan, Italy

**Barbara LeRoy, PhD**

Developmental Disabilities Institute, Wayne State University, Detroit, Michigan, USA

**Jennifer Mendez, PhD**

School of Medicine, Wayne State University, Detroit, Michigan, USA

**Michelle Putnam, PhD**

School of Social Work, Simmons College, Boston, USA

**Andria Spindel, MSW**

March of Dimes Canada, Toronto, Canada
